# Supplementary material for: Extract from Black Soybean Cultivar A63 Extract Ameliorates Atopic Dermatitis-like Skin Inflammation in an Oxazolone-Induced Murine Model
Source: Molecules. 2022 Apr 25;27(9):2751. doi: 10.3390/molecules27092751 (PMC9104407; doi:10.3390/molecules27092751)
Supplement: Supplementary file 1 [file molecules-27-02751-s001.zip › molecules-1670887-supplementary.pdf]

Korean black soybean A63 extract ameliorates atopic dermatitis-like skin inflammation in an oxazolone-induced murine model.

Banzragch Dorjsembe [1,4 +], Joon Won Lee[2,+], Yongsoo Choi[2,*], and Jin Chul Kim[3,*]

[1] Smart Farm Research Center, Korea Institute of Science and Technology, Gang neung, 25451, Republic of Korea.

[2] Natural Product Research Center, Korea Institute of Science and Technology, Gangneung, 25451, Republic of Korea.

[3] Natural Product Informatics Research Center, Korea Institute of Science and Te chnology, 25451, Gangneung, Republic of Korea

[4] KIST School, Korea University of Science and Technology, Daejeon, 34113, R epublic of Korea

Supplementary material

Fig.S1. DPPH assay (A) and Liver cell protective assay (B) result.

Plant sampling and DPPH and liver cell protective assay were performed according to previou s method of Xue et al (2017). Sample concentration of black soybean extracts were 0.25 mg/m l for DPPH assay and 0.1 mg/ml for Liver cell protective assay.

Fig.S2. UV Chromatogram of major components of A63 and ST.

HPLC and UV detector used for isoflavone detection. Wavelength for isoflavone was 254 nm. Detected compounds were numbered as following:

1. Daidzin, 2.6-Malonyldaidzin, 3.6-Malonylgenistin, 4. Daidzein, 5. Genistin.

Fig.S3. Full length western blot images of Fig.5

Fig.S4. Full length western blot images of Fig.6

Table S1. List of Primers.

Table S2. Comparing of dietary contents in various species of soybean extract

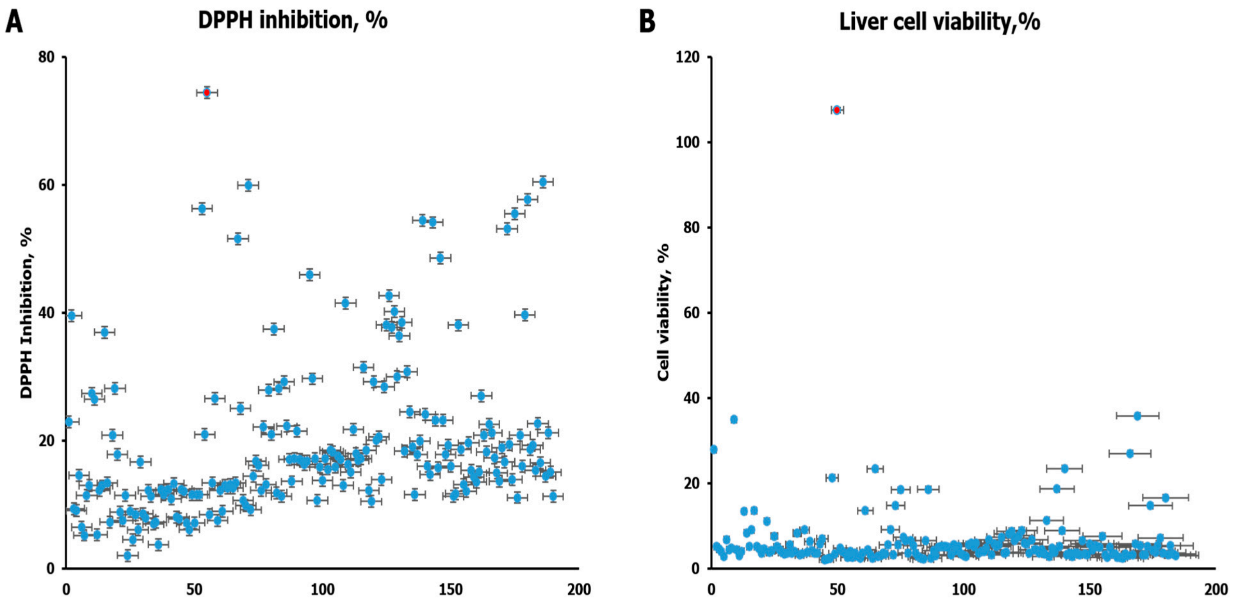

Figure S1. DPPH assay (A) and Liver cell protective assay (B) resu
lt.

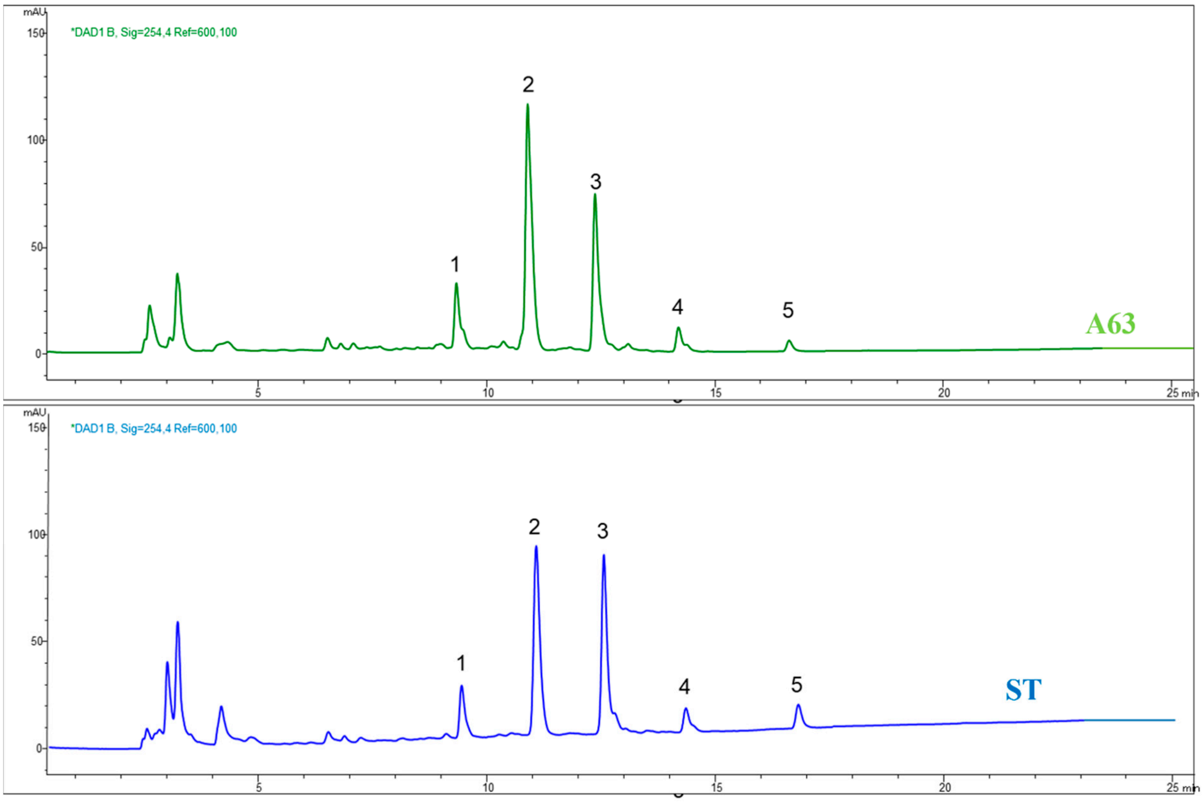

Figure S2. UV Chromatogram of major components of A63 and S
T.

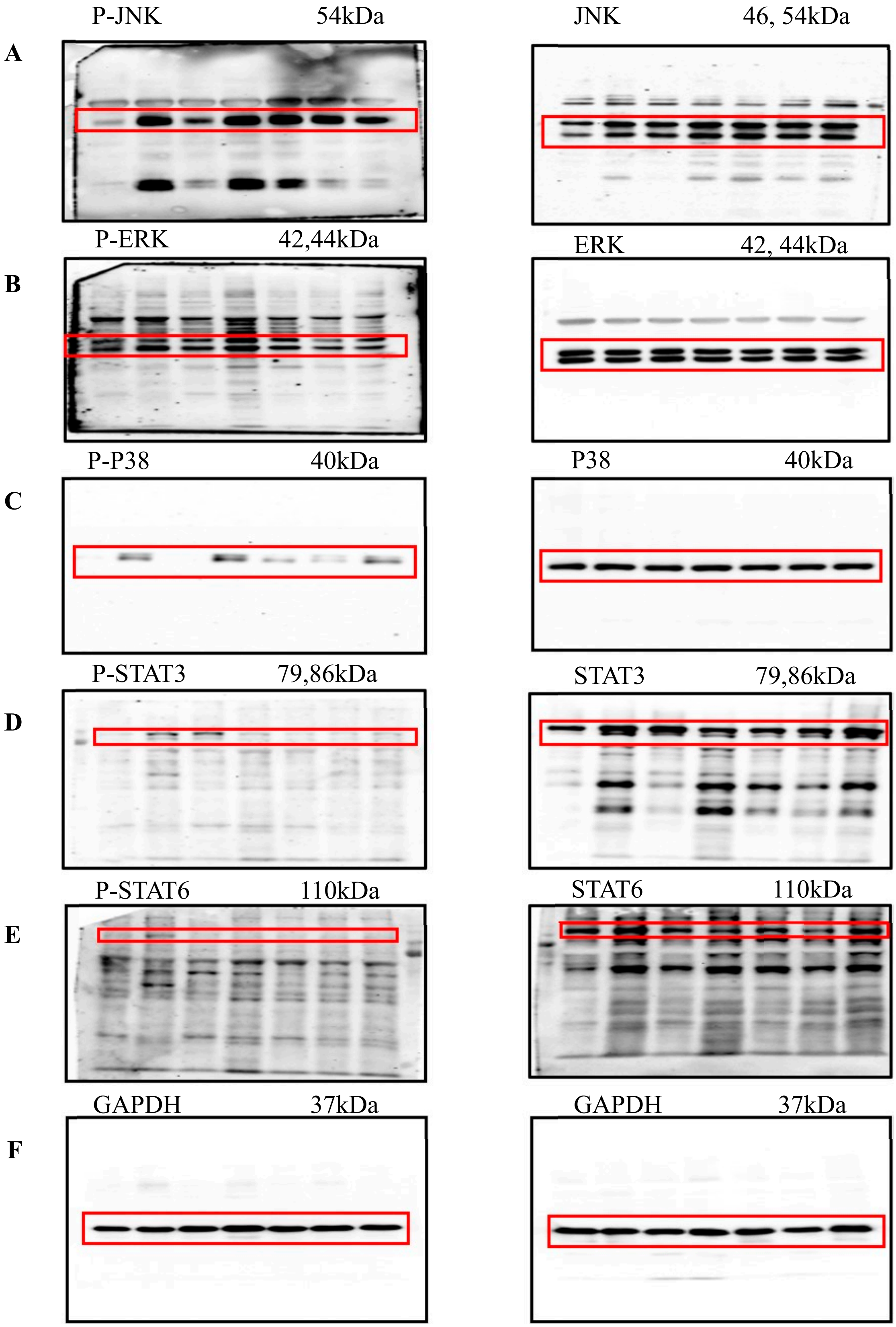

Figure S3. Full length western blot images of Figure 5.

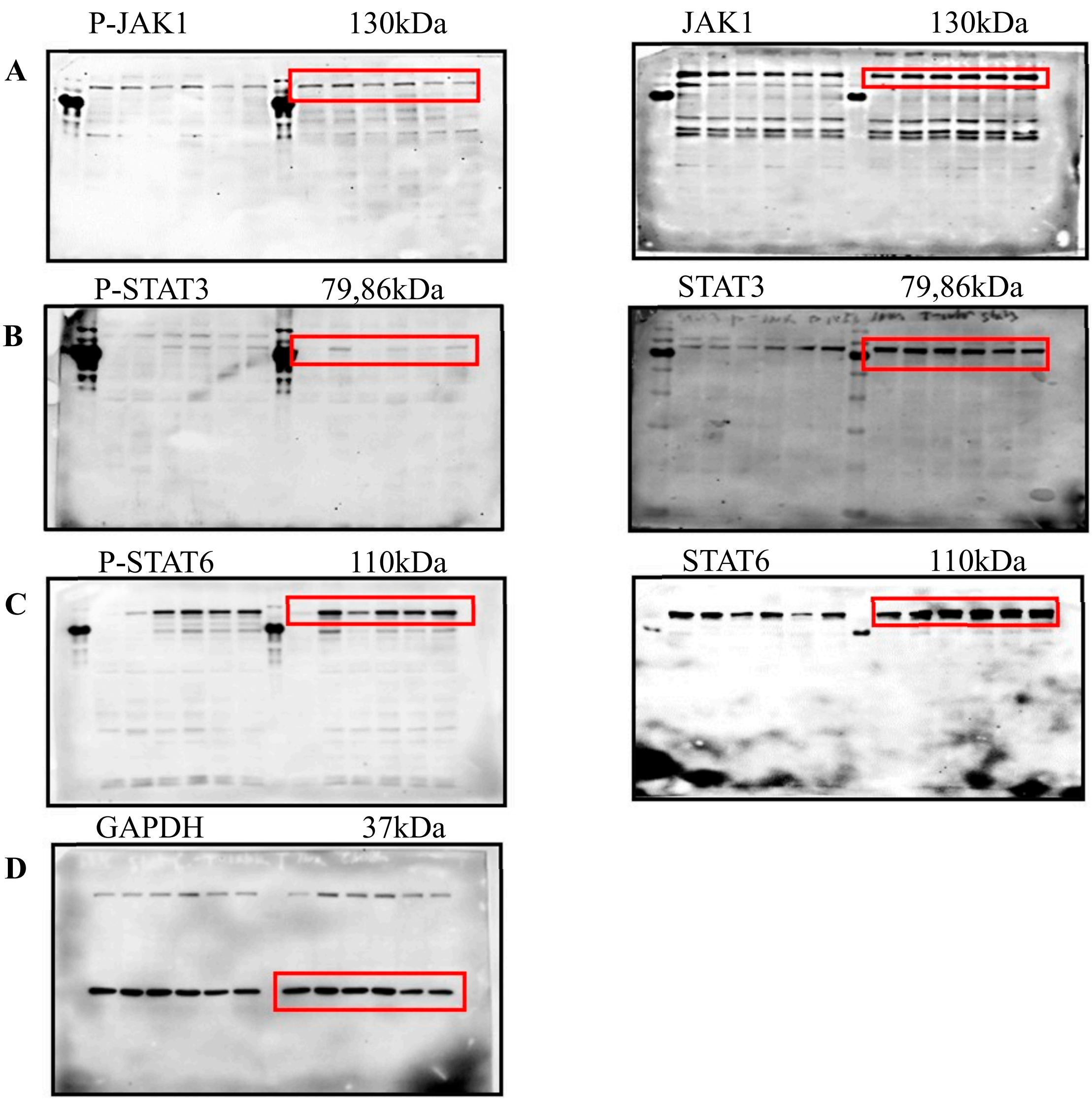

Figure S4. Full length western blot images of Figure 6.

Table S1. List of Primers.

Primer name	Sequence
M_IL-4_F	ATGGATGTGCCAAACGTCCT
M_IL-4_R	AAGCACCTTGGAAGCCCTAC
M_IL-5_F	CGTGGGGGTACTGTGGAAAT
M_IL-5_R	AATCCAGGAACTGCCTCGTC
M_CCL17_F	AATGTAGGCCGAGAGTGCTG
M_CCL17_R	TGCCCTGGACAGTCAGAAAC
M_CCL26_F	GTCCTGCTGCCCTAATTTCA
M_CCL26_R	TCACTGGTGCAGCTCTTGTC
H_CCL26_F	CCTCCTGAGTCTCCACCTTG
H_CCL26_R	GGATGGGTACAGACTTTCTTGC
M_GAPDH_F	CATGGCCTTCCGTGTTCCTA
M_GAPDH_R	ACTTGGCAGGTTTCTCCAGG

Table S2. Comparing of dietary contents in various species of soybean extract

	Contents (mg/g)							
	Anthocyanins	Pro-anthocyanidins	Flavonols	Isoflavonols				
	*Cyanidin-o-glucoside	*Procyanidin B2	*Epicatechin	#Daidzin	#6-Malonyldaidzin	#6-Malonylgenistin	#Daidzein	#Genistin
SCEL-1 (A63)	4.99	0.41	13.4	1.25-1.36	5.74-6.38	3.45-3.70	0.27-0.53	0-0.16
Seritae (ST)	2.67	0.11	1.28	0.73-0.94	4.18-4.43	4.26-4.52	0.08-0.38	0.09-0.38

* Mass spectrometry analysis # High performance liquid chromatography analysis
